# Antioxidant Effects of Quercetin Nanocrystals in Nanosuspension against Hydrogen Peroxide-Induced Oxidative Stress in a Zebrafish Model

**DOI:** 10.3390/ph16091209

**Published:** 2023-08-25

**Authors:** Junjie Wang, Xinyue Xue, Xiaoqing Miao

**Affiliations:** 1 Marine College, Shandong University, Weihai 264209, China; 202000700243@mail.sdu.edu.cn (J.W.); 201900810249@mail.sdu.edu.cn (X.X.); 2 SDU-ANU Joint Science College, Shandong University, Weihai 264209, China

**Keywords:** quercetin, nanocrystals, oxidative stress, zebrafish, antioxidant effect, particle size effect

## Abstract

Quercetin, a flavonoid compound rich in hydroxyl groups, possesses antioxidant properties, whereas its poor water solubility limits its bioavailability. In pursuit of addressing the water solubility of quercetin and comprehending the impact of nanocrystal particle size on antioxidant efficacy, we prepared three different-sized quercetin nanocrystals, namely small (50 nm), medium (140 nm), and large (360 nm), using a nanosuspension method in this study. Within the in vitro setting, assessments employing solubility and radical scavenging assays revealed that quercetin nanocrystals displayed superior solubility (26, 21, and 13 fold corresponding to small, medium, and large particle sizes) and antioxidant performance compared to the coarse quercetin. Furthermore, quercetin nanocrystals of three particle sizes all demonstrated significant protection effects on the survival rate of H_2_O_2_-treated zebrafish at 72 h (77.78%, 73.33%, and 66.67% for small, medium, and large particle sizes, respectively), while the coarse quercetin group exhibited a low survival rate (53.3%) similar to the H_2_O_2_-treated group (47.8%). Moreover, all quercetin nanocrystals exhibited potent antioxidant capacity on both the antioxidants and enzymatic antioxidant system in H_2_O_2_-treated zebrafish to restore zebrafish to a normal state under oxidative stress. For instance, the levels of reactive oxygen species were reduced to 101.10%, 108.83%, and 109.77% of the normal levels for small, medium, and large particle-sized quercetin nanocrystals, respectively. In conclusion, quercetin nanocrystals demonstrated enhanced solubility, robust antioxidant capacity, and protective effects in zebrafish compared to coarse quercetin.

## 1. Introduction

Reactive oxygen species (ROS) are inevitable products of cellular oxidative metabolism and are generally maintained in a state of balance between pro-oxidant and antioxidant activity [1]. However, many environmental stimuli, including thermal factors, growth factors, and ultraviolet radiation, can cause excessive ROS, disrupting the redox balance and inducing cells to enter a state of oxidative stress [2]. Oxidative stress can lead to irreversible damage to cellular DNA, lipids, or proteins, resulting in severe consequences such as cell death, carcinogenesis, and aging [3]. An effective strategy to reduce oxidative stress in humans is to increase the intake of antioxidant phytochemicals [4].

Quercetin (QUE), a naturally occurring polyphenolic flavonoid found in fruits and vegetables, possesses significant antioxidant activity, whereas most commercially available synthetic antioxidants are toxic [5,6]. QUE is considered to be able to scavenge ROS and enhance the activity of antioxidant enzymes such as catalase (CAT) and glutathione peroxidase (GPx) levels [7]. However, the water solubility of QUE is very poor, which indirectly or directly leads to the low bioavailability of QUE in the human body, even below 1% [8]. Therefore, the low water solubility of QUE limits its medical and food applications. Various transport systems were reported to improve the stability, solubility, and bioavailability issues of QUE, such as liposomes [9], capsules [10], and functional packaging films [11]. However, the drug loading capacity of these transport systems is low, and they need carrier materials. In contrast, drug nanocrystals (NCs) consist of nearly 100% drug and do not require a carrier system, offering a higher safety profile [12]. At the same time, the high drug-carrying capacity allows them to achieve high drug concentrations when delivering drugs to or into cells [13]. However, in some cases, nanomaterials have demonstrated toxicity in vitro and in vivo [14,15]. Therefore, it is crucial to thoroughly understand the key aspects of NCs’ interaction with living systems and accurately compare the benefits and potential hazards associated with their use.

Zebrafish (*Danio rerio*), a vertebrate model representing intact biological individuals, has been widely adopted in recent years for oxidative stress research [16]. This species is highly reproductive and has had its whole genome sequenced, which aligns with the human genome [17]. In addition, the transparency of zebrafish embryos allows for the observation of drug effects on their development, and it also facilitates fluorescence observation through various routes [18]. For instance, hybrid NCs synthesized by aggregation-induced emission (AIE) can be used to characterize the transport and distribution of NCs in zebrafish. Currently, zebrafish have been used in UVB-induced [19], NaAsO_2_-induced [20], APPH-induced [21], and hydrogen peroxide-induced oxidative stress models [22]. Based on the pre-experiment results of oxidative stress, this study will use hydrogen peroxide to induce oxidative stress in zebrafish and validate the feasibility of using zebrafish to characterize the in vivo antioxidant effects of nanosized QUE.

Due to the small size of NCs, they can enter cells and interact with various cellular structures within their size range, thereby exerting specific effects. Some NCs were reported to have a size-dependent effect on tumor cell proliferation, adhesion, and migration [23]. In addition, different particle sizes are reported to affect the uptake and potential toxicity of particles [24]. Furthermore, since the pore size of the chorion canals in zebrafish was approximately 0.5–0.7 μm [25], it was necessary to fabricate particle sizes smaller than the pore size to assess the in vivo effect of QUE-NCs. Accordingly, three different-sized quercetin nanocrystals (QUE-NCs), namely small (50 nm), medium (140 nm), and large (360 nm), were prepared to investigate the size effects on the antioxidant properties of QUE-NCs.

This study focused on investigating the particle size effects of QUE-NCs prepared by a nanosuspension method on antioxidant capacity in vitro and in vivo. Firstly, the small-sized QUE-NCs showed superiority over large-sized NCs in vitro antioxidant and in aqueous solubility studies. In addition, we observed the transport of hybrid QUE-NCs synthesized by AIE in zebrafish embryos, where small-sized QUE-NCs exhibited better permeability to the chorion. However, there was an adverse effect of small-sized QUE-NCs in vivo, lowering the heart rate of zebrafish even at low concentrations. In studying antioxidants and the enzymatic antioxidant system in zebrafish, QUE-NCs of all particle sizes displayed significant protective abilities, even restoring zebrafish to a normal state under oxidative stress. The findings suggested that nanocrystallization of QUE could effectively exploit the antioxidant effects and be important in developing drug-load nanoparticles for clinical application. Furthermore, it was necessary to consider safety issues further and find a balance between the positive and negative effects of particle size in vivo.

## 2. Results and Discussion

### 2.1. Characterization of QUE-NCs

#### 2.1.1. Size, Morphology, and Stability of QUE-NCs

**Figure 1:**
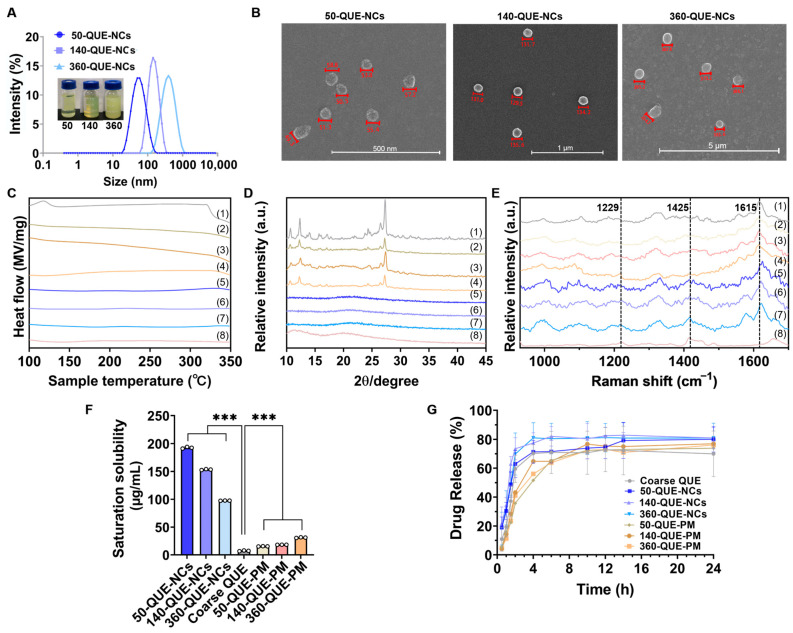
(**A**) The size distributions and photographs of 50-QUE-NCs, 140-QUE-NCs, and 360-QUE-NCs; (**B**) SEM morphology of 50-QUE-NCs, 140-QUE-NCs, and 360-QUE-NCs; (**C**) DSC curves, (**D**) XRD profiles and (**E**) Raman spectra of (1) QUE, (2) 50-QUE-PM, (3) 140-QUE-PM, (4) 360-QUE-PM, (5) 50-QUE-NCs, (6) 140-QUE-NCs (7) 360-QUE-NCs and (8) PVP; (**F**) saturation solubility of 50-QUE-NCs, 140-QUE-NCs, 360-QUE-NCs, coarse QUE, 50-QUE-PM, 140-QUE-PM, and 360-QUE-PM (The specific data are presented as white circles in the figure; *** *p* < 0.001); (**G**) drug release curves.

QUE-NCs of three particle sizes, namely small, medium, and large, were successfully prepared using the anti-solvent precipitation method. The mean particle sizes of the QUE-NCs were 51.3 ± 0.1 nm, 139.0 ± 0.6 nm, and 358.9 ± 4.7 nm (Figure 1A), with corresponding PDIs of 0.184 ± 0.016, 0.068 ± 0.01, and 0.168 ± 0.022, and zeta potentials of −16.4 ± 2.3 mV, −15.1 ± 0.7 mV, and −13.6 ± 0.6 mV, respectively. All PDIs of the QUE-NCs were less than 0.2, indicating good homogeneity in the suspensions. Furthermore, the particle sizes of the QUE-NCs observed in the SEM images (Figure 1B) were consistent with the dynamic light scattering (DLS) measurements for each particle size. Additionally, the QUE-NCs displayed a spherical-like morphology as other reported nanomaterials [26].

The three nanosuspensions showed good storage stability over five days, with slight increases in particle sizes and PDI observed (Table S1). On approximately the sixth day, the nanosuspensions started to agglomerate and form precipitates, possibly due to the high surface energy and Ostwald ripening effect of the NCs [27]. Furthermore, the sharp decrease in zeta potential on the sixth day may have also contributed to the precipitation (Table S1).

#### 2.1.2. Solid State Characterization of QUE-NCs

The thermal behaviors, crystalline properties, and chemical properties of QUE-NCs in the solid state were explored to assess the effects of nanocrystal formulation processes. The DSC curves of 50-QUE-NCs, 140-QUE-NCs, 360-QUE-NCs, coarse QUE, physical mixture, and PVP are shown in Figure 1C. The endothermal peak of QUE was observed at 321.12 °C, and the peak at 109.61 °C represented the exothermic peak due to adsorbed water in the sample. PVP did not exhibit a typical endothermal peak. The curves for the physical mixtures were equivalent to the superimposed QUE and PVP. However, the endothermal peaks of the three QUE-NCs were significantly absent, indicating the formation of the amorphous state of QUE-NCs. One possible reason for the amorphous state of QUE-NCs was that PVP, an amorphous material, suppressed the recrystallization of QUE by forming a high-energy amorphous state during the nanocrystallization process [28].

To further investigate the crystalline properties of QUE-NCs, the crystallinities of 50-QUE-NCs, 140-QUE-NCs, 360-QUE-NCs, coarse QUE, physical mixtures, and PVP were detected using XRD (Figure 1D). QUE exhibited prominent crystal characteristic diffraction peaks at 2θ = 10.70°, 12.36°, 24.32°, 26.60°, and 27.33°, which matched PDF-2 (43–1695) and indicated the well-defined initial crystalline state of QUE. The patterns of the physical mixtures were similar to that of QUE, indicating no changes in the crystalline phase structure. In contrast, the spectra of QUE-NCs decreased the intensity of peaks and showed broad and diffuse patterns without obvious crystal characteristic diffraction peaks, confirming their essentially amorphous form [8,29]. Similar findings for amorphous drugs were also reported by Fan et al. [30].

The Raman spectra of 50-QUE-NCs, 140-QUE-NCs, 360-QUE-NCs, coarse QUE, physical mixtures, and PVP are illustrated in Figure 1E. The most intense characteristic spectral band of QUE was observed at 1615 cm^−1^, corresponding to C-O and C=C stretching in the A, B, and C rings [31,32]. 50-QUE-NCs, 140-QUE-NCs, 360-QUE-NCs, and physical mixtures exhibited similar most intense characteristic spectral bands of QUE. However, the spectral bands of the three QUE-NCs at 1425 cm^−1^ (3,5-OH and C-H in-plane bending and C=O stretching) and 1229 cm^−1^ (C-H in-plane bending) differed from QUE [31,33], which can be attributed to the superimposed effect of PVP and QUE. These results indicate that the nanocrystal formulation processes had no influence on the chemical structure of QUE and successful preparation of QUE-NCs was achieved.

### 2.2. Solubility Study

The saturation solubility of 50-QUE-NCs, 140-QUE-NCs, 360-QUE-NCs, QUE, and physical mixtures in water is shown in Figure 1F. The aqueous solubility of QUE was only 7.32 μg/mL, indicating its practical insolubility in water. The physical mixtures exhibited a slight increase in the solubility of QUE (15.43 μg/mL, 18.28 μg/mL, and 31.11 μg/mL for 50-QUE-PM, 140-QUE-PM, and 360-QUE-PM, respectively). Additionally, the fabricated NCs significantly improved the aqueous solubility of QUE, with enhancements of 26.3, 21.0, and 13.3 fold for 50-QUE-NCs, 140-QUE-NCs, and 360-QUE-NCs, respectively. This phenomenon can be attributed to the wetting action, surfactant hydrophilicity, and simultaneous reduction of particle sizes [34].

### 2.3. In Vitro Drug Release Study

The release curves of coarse QUE, 50-QUE-NCs, 140-QUE-NCs, 360-QUE-NCs, and physical mixtures in vitro are presented in Figure 1G. The percentage of QUE released within 24 h from coarse QUE, 50-QUE-NCs, 140-QUE-NCs, and 360-QUE-NCs was 69.96%, 79.95%, 80.95%, and 80.89%, respectively. No apparent differences were found among the three particle sizes (comparisons of each size, *p*-values > 0.05). From the release profiles, it can be observed that QUE-NCs exhibited better cumulative release than coarse QUE, and the cumulative release of physical mixtures was in between coarse QUE and QUE-NCs. Moreover, the dissolution rates of QUE-NCs were faster than those of coarse QUE and physical mixtures during the first four hours, which can be attributed to the small size and large specific surface areas of the NCs [35].

### 2.4. The Radical Scavenging Ability of QUE-NCs In Vitro

**Figure 2:**
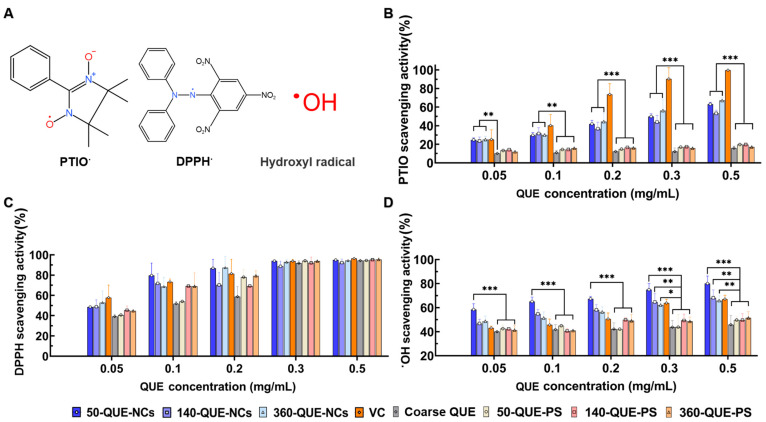
(**A**) Chemical structure formulas of PTIO, DPPH, and hydroxyl radical. The radical scavenging abilities of coarse QUE, physical mixtures, and QUE-NCs with VC as a control group: (**B**) PTIO radical, (**C**) DPPH radical and (**D**) hydroxyl radical. (* *p* < 0.05, ** *p* < 0.01, and *** *p* < 0.001).

The following radical scavenging assays were performed to analyze the antioxidant effect of QUE by assessing the capacity of QUE-NCs to scavenge reactive oxygen species (ROS) and reactive nitrogen species (RNS). VC, a common non-enzymatic antioxidant known for its free radical scavenging activity [36], was used as a positive control to compare the effects of QUE-NCs. The chemical structure formulas of PTIO, DPPH, and the hydroxyl radical were demonstrated in Figure 2A.

The PTIO radical was found to be a new, stable, oxygen-centered radical for evaluating ROS scavenging levels, and it could be easily detected due to its extensive π-π conjugative system with long wavelength absorbance [37]. The scavenging ability of coarse QUE, physical mixtures, and QUE-NCs on the PTIO radical was shown in Figure 2B, compared with VC as the standard control. The PTIO scavenging ability of coarse QUE and physical mixtures varied slightly with concentration, which may be attributed to their low aqueous solubility. As a result, only a small amount of quercetin reacts with PTIO, resulting in a weak scavenging capacity. In contrast, it was evident that both QUE-NCs and VC showed concentration-dependent increases in PTIO radical scavenging capacity, with marginal differences observed among the QUE-NCs of three particle sizes.

The DPPH radical was a reactive hydrogen acceptor with a single electron at the nitrogen atom (Figure 2A), which can be used to determine the scavenging activity of reactive nitrogen species (RNS) [38]. As depicted in Figure 2C, the DPPH scavenging effect of three-sized QUE-NCs demonstrated a positive correlation with increasing concentration. All samples exhibited more than 90% scavenging effect at a concentration of 0.5 mg/mL. Notably, the DPPH radical scavenging ability of 50-QUE-NCs was even better than that of VC in the concentration range of 0.1 mg/mL to 0.3 mg/mL. Furthermore, the DPPH scavenging ability of coarse QUE and physical mixtures also varied with concentrations, likely due to the fact that DPPH was dissolved in organic solvents, and coarse QUE exhibited good solubility in organic solvents as well. Thus, both QUE-NCs and coarse QUE demonstrated effective reactivity towards DPPH.

The hydroxyl radical was a representative reactive oxygen species, capable of readily reacting with most cellular molecules, thereby causing severe damage to biomacromolecules [39]. The results of the hydroxyl radical scavenging assay by inhibiting the Fenton reaction are illustrated in Figure 2D. It is evident that Que exhibits good scavenging ability for hydroxyl radicals, and nanocrystallization of QUE enhances this ability, particularly with small particle size QUE-NCs. However, coarse QUE was limited by its solubility, and the scavenging gap between coarse QUE and QUE-NCs became increasingly significant with increasing concentrations.

All of the results mentioned above indicated that QUE-NCs have a significant effect on scavenging reactive oxygen species (ROS) and reactive nitrogen species (RNS), surpassing coarse QUE in PTIO and hydroxyl radical scavenging. Furthermore, the scavenging abilities of QUE-NCs with three different particle sizes showed slight differences, with small particle-sized NCs exhibiting greater scavenging abilities in most cases. The possible reasons for these results were that the successfully prepared QUE-NCs improved the water solubility of QUE, and small particle size NCs had a larger specific surface area, resulting in more phenolic hydroxyl groups being exposed to the spherical surface [30,40].

### 2.5. Effects of QUE-NCs in Zebrafish In Vivo

#### 2.5.1. Toxicity of QUE-NCs on the Development of Zebrafish Embryos

**Figure 3:**
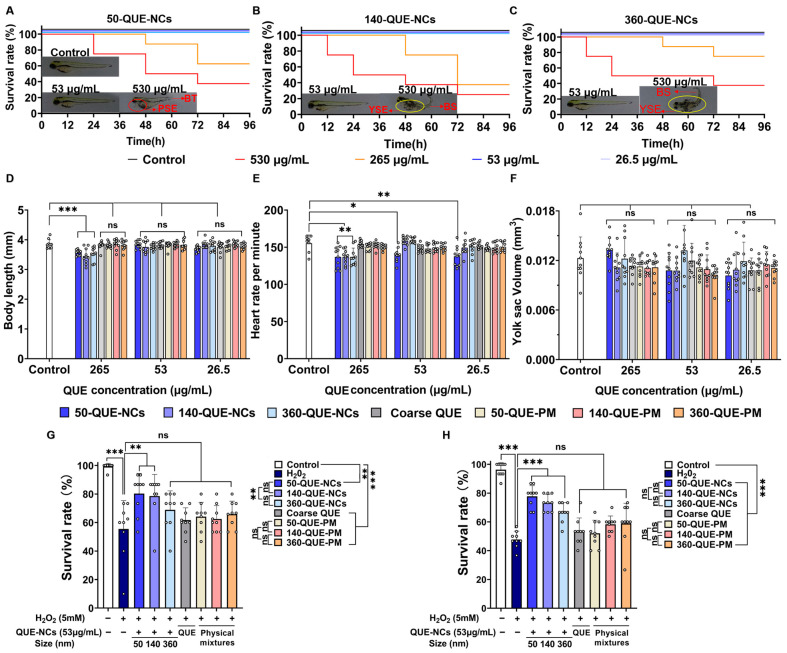
Survival rates of zebrafish incubated in (**A**) 50-QUE-NCs, (**B**) 140-QUE-NCs, and (**C**) 360-QUE-NCs and malformations of zebrafish (BS, bend spine; BT, bend tail; YSE, yolk sac edema; PSE, pericardial sac edema); (**D**) body length of zebrafish; (**E**) heart rate per minute of zebrafish; (**F**) yolk sac volume of zebrafish; (**G**) survival rates of zebrafish treated with 5 mM-H_2_O_2_ at 24 h; (**H**) survival rates of zebrafish treated with 5 mM-H_2_O_2_ at 72 h. The specific data are presented as white circles in the figures; ‘ns’ non-significant, * *p* < 0.05, ** *p* < 0.01, and *** *p* < 0.001.

To eliminate the potential toxicity of QUE-NCs on zebrafish survival and related development after the addition of hydrogen peroxide, we evaluated several phenotypic parameters on zebrafish at different concentrations to identify the appropriate drug concentration. The phenotypic parameters included survival rate, heartbeat per minute, body length, and yolk sac volume. As shown in Figure 3A–C, zebrafish embryo mortality significantly increased with time at concentrations of 265 μg/mL and 530 μg/mL, regardless of nanoparticle size. In contrast, coarse QUE and physical mixtures showed minimal toxic effects on the survival rate and physiological characteristics of zebrafish at all concentrations, possibly due to their low water solubility (Figure 3D–F and Figure S1). Additionally, surviving larvae exhibited malformations such as yolk sac edema, pericardial sac edema, bent tail, and bent spine when exposed to concentrations of 265 μg/mL and 530 μg/mL. Furthermore, surviving zebrafish exposed to a concentration of 265 μg/mL showed a significant reduction in body length (Figure 3D). These detrimental effects on zebrafish indicate a dose-dependent response to QUE-NC exposure, potentially due to QUE-NCs impacting nutrient uptake from the yolk sac, leading to increased lethality and deformities [25]. Nevertheless, it did not imply that QUE-NCs could be considered as exhibiting hormesis since the biphasic dose–response phenomenon did not occur. In addition, as shown in Figure 3E, the heart rate of zebrafish embryos was significantly suppressed when exposed to 50-QUE-NCs at all concentrations compared to the control group. A decreased heart rate was indicative of heart failure [41], and this could be attributed to the higher permeability of small NCs in zebrafish [42], which subsequently has a detrimental effect on atrial contraction [43]. Therefore, it is necessary to consider safety issues further and find a balance between the positive and negative effects of particle size.

However, QUE-NCs at all concentrations showed no significant effect on the yolk sac volume of zebrafish embryos compared to the control group (Figure 3F). Zebrafish incubated with NCs at a high concentration of 265 μg/mL exhibited constraints in both heart rate and body length, consistent with other reported toxicities of drugs on zebrafish (Due to high lethality and deformity rates, the zebrafish exposed to a concentration of 530 μg/mL were not studied) [44]. In contrast, zebrafish cultured at a concentration of 53 μg/mL showed minimal effects on survival rate, heart rate, and body length, making it suitable for subsequent antioxidant assays. Fortunately, QUE-NCs at this concentration exhibited prominent protection and improved the survival rates of zebrafish at 24 h and 72 h under hydrogen peroxide exposure (Figure 3G,H). However, there was no significant difference in survival rate between zebrafish exposed to coarse QUE and the H_2_O_2_ group. Additionally, 50-QUE-NCs showed better protection at 24 h, which could be attributed to the greater permeability of small-sized NCs to the chorion of zebrafish during the initial period.

#### 2.5.2. Transport of QUE-NCs in Zebrafish Embryos In Vivo

The zebrafish model has recently been utilized in toxicology and transport studies due to its chorion, a peptide membrane structure acting as a biological barrier to protect itself during early developmental stages [45,46]. Therefore, the permeability of compounds in zebrafish embryos was believed to be directly associated with their ability to enter through the chorion [47]. To investigate the permeability of nanoparticles with different particle sizes on the zebrafish chorion, we conducted the following visualization experiments. However, in the study of HPS-QUE-NCs transport in zebrafish, coarse QUE was challenging to label and observe due to the poor fluorescence of QUE itself [48] and its low solubility in water. As a result, it was not used as a control in the experiment.

**Figure 4:**
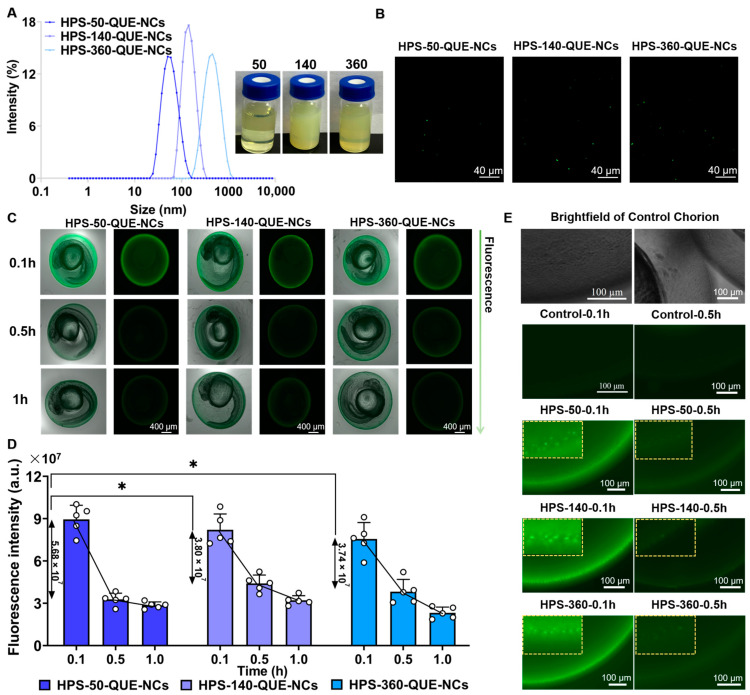
(**A**) The size distributions and photographs of HPS-50-QUE-NCs, HPS-140-QUE-NCs, and HPS-360-QUE-NCs; (**B**) fluorescence images of HPS-QUE-NCs (the green dotted fluorescence was generated through the excitation of HPS, which was encapsulated by QUE-NCs); (**C**) brightfield and fluorescence images of zebrafish embryos from 0.1 h to 1 h (the arrow on the right showed the change in fluorescence intensity); (**D**) the fluorescence intensity variation of zebrafish embryos, during one-hour continuous administration of HPS-QUE-NCs (the numbers located on the vertical arrows were the fluorescence changes of zebrafish embryos from 0.1 h to 0.5 h); (**E**) the local images of zebrafish embryonic chorion from 0.1 h to 0.5 h (inside yellow dotted boxes were re-enlarged image; the left was magnified by 25 fold, and the right was magnified by 9 fold). The specific data are presented as white circles in the figure; * *p* < 0.05.

The fluorophores (AIE) were entrapped in hybrid QUE-NCs using the method described by Li, YX et al. (2022) [49]. The mean particle sizes of HPS-QUE-NCs were 58.2 ± 0.7 nm, 164.4 ± 0.5 nm, and 393.2 ± 1.4 nm, with PDIs of 0.232 ± 0.006, 0.105 ± 0.03, and 0.174 ± 0.016, and zeta potentials of −15.4 ± 1.1 mV, −12.7 ± 0.3 mV, and 9.9 ± 0.5 mV, respectively. These sizes indicated that the hybrid QUE-NCs were uniformly enlarged, confirming the successful coating of HPS-QUE-NCs (Figure 4A). Previous studies have reported the ability of NCs to penetrate the zebrafish chorion and track their transport within zebrafish [50]. Therefore, the hybrid QUE-NCs (Figure 4B) were considered capable of indicating the absorption and metabolism of QUE-NCs in vitro through fluorescence variation. As shown in Figure 4C, HPS-50-QUE-NCs, HPS-140-QUE-NCs, and HPS-360-QUE-NCs were used to assess the uptake and transport of QUE-NCs in zebrafish embryos. The fluorescence intensity on the chorionic membrane of zebrafish embryos decreased over time during the first hour of continuous administration. It was considered the continuous uptake of hybrid QUE-NCs by zebrafish embryos and the dynamic transport process within the chorion, as depicted in Figure 4E. Additionally, Figure 4D demonstrates that the fluorescence intensity of zebrafish embryos incubated in the 50-QUE-NCs suspension sharply declined within 0.5 h of initial continuous administration, indicating that QUE-NCs with smaller particle sizes exhibited greater permeability compared to medium and large sizes.

**Figure 5:**
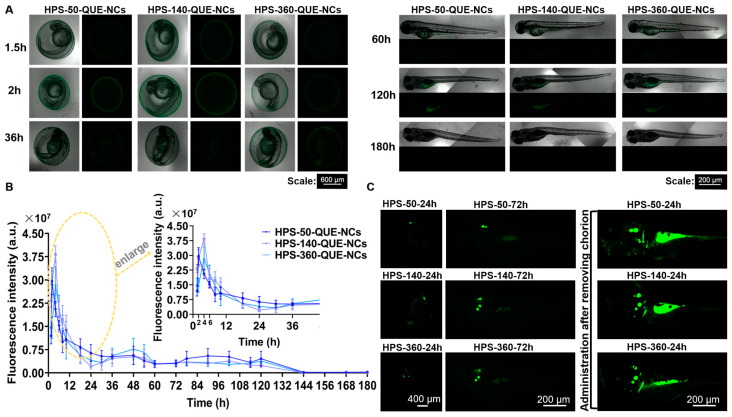
(**A**) Brightfield and fluorescence images of zebrafish embryos from 1.5 h to 180 h, after rinsing by E3 medium and incubating in E3 medium; (**B**) the fluorescence intensity variations of zebrafish embryos after stopping administration and local enlargement images (indicating the area outlined by a yellow dotted line) from 0–48 h; (**C**) distributions of QUE-NCs in zebrafish (Clear fluorescence observed at the otolith of zebrafish after prolonging the administration time to 24 h, showing the partial distribution of QUE in the zebrafish. It became more apparent when administrated after the zebrafish removed the chorion).

One hour after administration, zebrafish embryos were rinsed and transferred to the E3 medium to observe the metabolism of QUE-NCs in zebrafish. As shown in Figure 5A,B, the fluorescence of the three-size NCs rose and fell within six hours after the termination of the dosage. One possible explanation was that the NCs initially adsorbed to the chorionic membrane [24], then gradually passed through the membrane and accumulated in the zebrafish, resulting in an increase in fluorescence intensity. The fluctuations in fluorescence observed in zebrafish 48 h after the end of administration were attributed to the transport of NCs throughout the body via blood circulation in the arteries due to the convergence of the bilateral aortic arches in zebrafish during the hatching period, forming a single artery just below the eye, which penetrated inside the head to form the carotid artery in vivo [51]. There were no statistically significant differences observed between the NCs of the three particle sizes (comparisons of each size, *p*-values > 0.05). Additionally, during the transport study of QUE-NCs, an interesting phenomenon was discovered where the distribution of QUE-NCs partially occurred in the otolith of zebrafish (Figure 5C). Furthermore, this phenomenon was particularly pronounced in zebrafish larvae when administered after hatching, as nanoparticles were more easily ingested through the mouth [25].

#### 2.5.3. Inhibitory Effects of QUE-NCs on ROS Generation, Lipid Peroxidation, and Cell Death in Zebrafish Embryos

**Figure 6:**
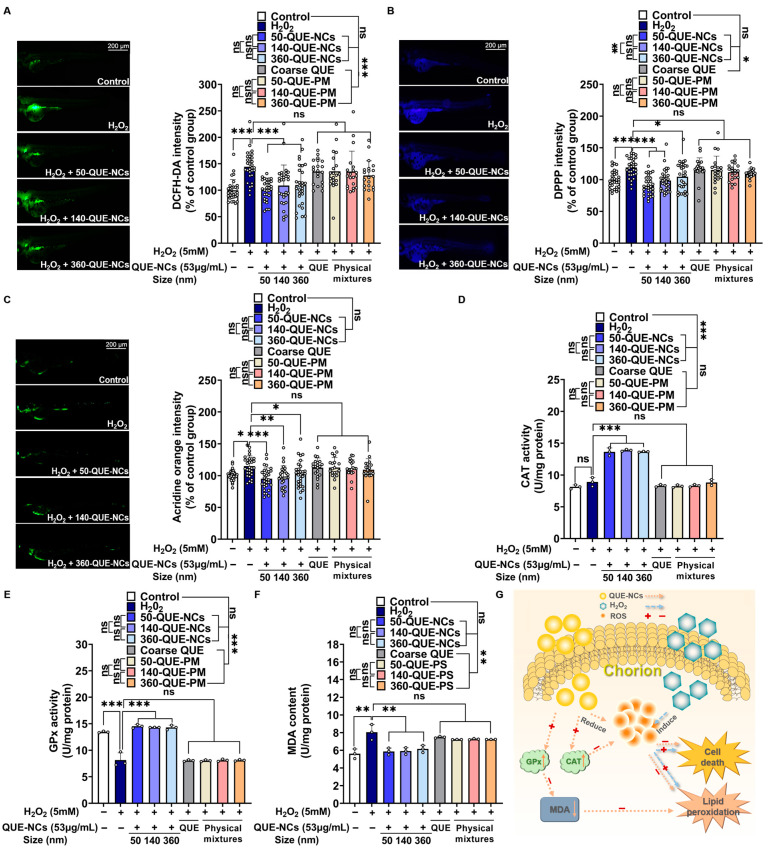
Protective effects of QUE-NCs on H_2_O_2_-induced oxidative stress, lipid peroxidation, and cell death in zebrafish. The embryos were treated with 5 mM H_2_O_2_ and preconditioned with QUE-NCs. After incubation, the zebrafish were stained with DCFH-DA (**A**), DPPP (**B**), and AO (**C**). The fluorescence colors of DCFH-DA, DPPP, and AO at specific excitation wavelengths were green, blue, and green, respectively. The fluorescence intensity of the zebrafish was quantified using Image J (compared to the control group). (**D**) CAT activity of zebrafish; (**E**) GPx activity of zebrafish; (**F**) MDA content of zebrafish; (**G**) the process of QUE-NCs reducing the damage caused by oxidative stress in zebrafish. The specific data are presented as white circles in the figures; ‘ns’ non-significant, * *p* < 0.05, ** *p* < 0.01, and *** *p* < 0.001.

Oxidative damage was mainly caused by ROS, and QUE was considered capable of removing ROS. To examine the inhibitory effect of QUE-NCs on ROS generation, the fluorescent probe DCFH-DA was utilized. As shown in Figure 6A, nearly no fluorescence was observed in the control group of zebrafish after culturing with DCFH-DA. However, the fluorescence intensity of the 5 mM H_2_O_2_-treated group was dramatically enhanced to 144.30%, indicating the excessive generation of ROS under hydrogen peroxide-induced stress. Conversely, the groups preconditioned with QUE-NCs exhibited a noticeable reduction in the newly generated ROS. Specifically, 50-QUE-NCs, 140-QUE-NCs, and 360-QUE-NCs lowered its level to 101.10%, 108.83%, and 109.77%, respectively.

Lipid peroxidation not only converts ROS into non-radical lipid breakdown products but also amplifies the damage caused by ROS through chain or chain-branching reactions [52]. Therefore, to investigate whether QUE-NCs prevented lipid peroxidation and further decreased ROS damage, DPPP was used to detect the variations in fluorescence in zebrafish. As depicted in Figure 6B, the presence of DPPP in the 5 mM H_2_O_2_-treated group showed additional blue fluorescence, indicating the production of lipid peroxidation. When treated with QUE-NCs, the blue fluorescence in zebrafish exhibited a sharp decline, and the three particle-sized NCs (50-QUE-NCs, 140-QUE-NCs, and 360-QUE-NCs) each dropped to 90.46%, 98.55%, and 106.21%.

One potential mechanism for the protective effect of QUE-NCs was that QUE could suppress the oxidative damage caused by ox-LDL, thereby preventing programmed cell death or apoptosis [53]. To investigate the protective effect of QUE-NCs against H_2_O_2_-induced oxidative stress, we assessed cell death by measuring the fluorescence intensity of acridine orange in zebrafish. As illustrated in Figure 6C, the fluorescence intensity of the 5 mM H_2_O_2_-treated group was significantly increased in zebrafish compared to the control group. By adding QUE-NCs, the cell death of zebrafish exposed to H_2_O_2_ was evidently reduced, with 50-QUE-NCs exhibiting a great protective effect with a relative cell death rate of 95.12%. This effect was followed by 140-QUE-NCs and 360-QUE-NCs, with 98.12% and 102.73% rates, respectively. However, there were no statistically significant differences between the NCs of the three particle sizes (comparisons of each size, *p*-values > 0.05). Additionally, neither the coarse QUE-treated group, nor the physical mixtures treated group exhibited differences compared to the H_2_O_2_-treated group in terms of ROS generation, lipid peroxidation production, and cell death. The results suggested that the oxidative damage could not be effectively inhibited in zebrafish when treated with coarse QUE and physical mixtures (the staining photographs of zebrafish from the coarse QUE, 50-QUE-PM-, 140-QUE-PM-, and 360-QUE-PM-treated groups are shown in Figure S2).

#### 2.5.4. Effects of QUE-NCs on the Activity of Antioxidant Enzymes in Zebrafish

Most of the ROS produced in the cells were scavenged by their own antioxidant system. In the enzymatic antioxidant defense system, CAT and GPx synergistically promoted the breakdown of hydrogen peroxide [2]. In contrast, MDA was the oxidation product of lipid peroxidation caused by free radical actions, which could aggravate cell damage [54]. Therefore, the content of CAT and GPx were essential parameters reflecting the potential antioxidant capacity, and the MDA content was also a parameter indirectly reflecting the degree of peroxidative damage. As illustrated in Figure 6D, the CAT activity of the control group was 8.13 U/mg protein, while 50-QUE-NCs, 140-QUE-NCs, and 360-QUE-NCs showed CAT activities of 13.65 U/mg protein, 13.89 U/mg protein, and 13.60 U/mg protein, respectively. These results indicated that the CAT activities of each QUE-NC were increased to 167.90%, 170.85%, and 167.28% compared to the control group. The GPx activity of the 5 mM H_2_O_2_-treated group notably decreased compared to the control group (Figure 6E), but this reduction could be prevented, and the activities could be restored to 108.86%, 107.25%, and 107.10% of the control group when incubated with 50-QUE-NCs, 140-QUE-NCs, and 360-QUE-NCs. At the same time, the MDA content of the 5 mM H_2_O_2_-treated group remarkably increased (Figure 6F), but it could also be reduced by QUE-NCs, with the MDA content returning to 104.71%, 105.46%, and 109.76% of the control group, respectively. However, no statistically significant differences were found between the QUE-NCs of the three particle sizes (comparisons for each size, *p*-values > 0.05). Furthermore, although the coarse QUE and physical mixture groups slightly enhanced antioxidant enzyme activity, they did not differ significantly from the H_2_O_2_-treated group.

Production of excess ROS was considered one of the biomarkers of oxidative stress [55]. In this research, the zebrafish treated with 5 mM H_2_O_2_ exhibited significant ROS production, which was intuitively observed through DCFH-DA dyeing and fluorescence quantification calculations (Figure 6A), indicating the occurrence of oxidative stress. This phenomenon could also be observed in AAPH-induced oxidative stress in zebrafish embryos [21]. Similarly, antioxidant enzymes such as CAT and GPx were described as the first line of defense for antioxidant protection and widely used as markers in assessing oxidative stress [54]. The study of the H_2_O_2_-treated group showed a slight increase in CAT activity (Figure 6D, no significant difference, *p*-value > 0.05). Zebrafish embryos developed an adaptive response to oxidative stress damage during development, increasing CAT activity [56]. Meanwhile, GPx activity sharply decreased to 68.92% of the control group, and the MDA content increased to 143.53% of the control group (Figure 6E,F), indicating that the zebrafish embryos were under oxidative stress. The excess ROS and the decrease in GPx activity likely led to MDA accumulation [57], as ROS promoted the formation of lipid radicals while GPx inhibited or reduced lipid peroxidation [58]. Ultimately, zebrafish apoptosis was visualized using acridine orange to represent the damage caused by oxidative stress (Figure 6C), where apoptosis dramatically increased in the 5 mM H_2_O_2_-treated group. However, regarding relative ROS production, lipid peroxidation, and apoptosis, zebrafish preconditioned with QUE-NCs were restored to almost normal levels compared with the 5 mM H_2_O_2_ group. Moreover, GPx activity and MDA content also returned to normal, except for a significant increase in CAT activity for the breakdown of excess H_2_O_2_.

The process of QUE-NCs reducing the damage caused by oxidative stress in zebrafish in the research was demonstrated in Figure 6G. QUE exhibited the ability to eliminate the intracellular accumulation of ROS and suppress the production of hydroxyl radicals by chelating metal ions [59]. Moreover, it reduced ox-LDL levels, effectively inhibiting lipid peroxidation [60]. Additionally, QUE could restore the activities of GPx and enhance CAT activity. These actions of quercetin in scavenging free radicals and boosting enzyme activity contributed to the reduction of cellular damage and consequent attenuation of apoptosis. Furthermore, the protective effects of quercetin against H_2_O_2_-induced apoptosis were reported to be mediated through the activation of the PI3K/Akt signaling pathway [61].

According to the reports by Jeon J S et al. [62], QUE could reduce intracellular ROS levels in HepG2 cells derived from liver cancer tissue. ROS concentrations were high in the zebrafish liver region, as illustrated in Figure 6A and reported by Köktürk M et al. [63], accurately revealing the elimination effect of QUE. Similarly, QUE could prevent an increase in ROS induced by H_2_O_2_ in hippocampal neurons [64]. Once excess ROS was cleared, mitochondrial and nuclear DNA damage at the cellular level, and lipid peroxidation, could be attenuated, thus preventing oxidative damage [65]. The oxidation product of lipid peroxidation, MDA, was accordingly reduced (Figure 6B,F). Additionally, QUE also had a catalytic effect on enzymatic antioxidant systems [66], as demonstrated by the promotion of CAT activity and the restoration of GPx activity in response to oxidative stress (Figure 6D,F). Ultimately, the effect of QUE led to a decrease in oxidative stress-induced apoptosis [67]. However, no significant differences were found in the antioxidant effects of QUE-NCs with three particle sizes during this zebrafish research. Possible reasons could be that there were no significant differences in the receptors that caused QUE-NCs of three particle sizes to affect endocytosis during recruitment and binding of cellular receptors, driving membrane wrapping [68], or the aggregation of QUE-NCs increased in the biological environment or upon internalization into cells. Therefore, there were no significant differences in metabolism with the three particle sizes in zebrafish [69], as also confirmed in Section 2.5.2. Moreover, the weak effects observed in zebrafish under oxidative stress for coarse QUE and physical mixture groups could be attributed to their low solubility in water compared to QUE-NCs, resulting in only a tiny amount of QUE being absorbed by zebrafish.

## 3. Materials and Methods

### 3.1. Materials

QUE with a purity higher than 97%, polyvinyl pyrrolidone (PVP), and 1,1,2,3,4,5-Hexaphenylsilacyclopenta-2,4-diene (HPS) were all purchased from Macklin (Shanghai, China). Ferrous sulfate, salicylic acid, absolute ethanol, and hydrogen peroxide were, respectively, bought from Meryer (Shanghai, China), Titan (Shanghai, China), Tianjin Yongda Chemical Reagent Co. (Tianjin, China), and Jinan Qiguang Technology Co. (Jinan, China). The chemical reagents used in this article were chromatographic and analytical.

### 3.2. Method of Fabricating QUE-NCs of Different Particle Sizes

QUE-NCs were prepared using the anti-solvent precipitation method [70]. Briefly, QUE-NCs were formed by injecting the organic phase (absolute ethanol) into the aqueous phase using a syringe at a flow rate of 6 mL/min (5 s) under stirring at 1000 rpm (ZNCL-S-5D, Shanghai, China). The detailed preparation conditions are shown in Table S2. The QUE-NCs suspensions were subjected to evaporation of the organic solvents using a rotary vacuum evaporator. The resulting solutions were used to evaluate the biological effects in vitro and in vivo.

### 3.3. Fabrication of QUE-NCs

#### 3.3.1. Investigation of the Size, Morphology, and Stability of QUE-NCs

The hydrodynamic size, polydisperse index (PDI, indicating dimensional homogeneity), and zeta potential of the NCs were measured using a Nano^®^ Zetasizer (V.7.13, Malvern Instruments, Worcestershire, UK). The exact morphologies of the NCs were determined using a Field Emission Scanning Electron Microscope (Nova Nano SEM 450, FEI, Hillsboro, OR, USA) [71]. The suspensions were stored at 4 °C for analysis and to investigate the long-term stability of the nanosuspension. The particle size, PDI, and zeta potential of QUE-NCs were measured on days 1, 2, 3, 4, 5, 6, 7, and 8.

#### 3.3.2. Differential Scanning Calorimetry

Differential scanning calorimetry (DSC) was performed to analyze the thermal properties of QUE, stabilizer, physical mixtures, and NCs of three different particle sizes. The physical mixtures had the same content of QUE and stabilizer as the NCs for each particle size and were named 50-QUE-PM (50 nm quercetin-physical mixture), 140-QUE-PM (140 nm quercetin-physical mixture), and 360-QUE-PM (360 nm quercetin-physical mixture), respectively. The powder samples were obtained by freezing the NCs at −80 °C for freeze-drying modification after evaporating the organic solvents from the QUE-NCs suspensions. Each sample was placed in a standard aluminum pan and heated at 10 °C/min from 100 to 350 °C (STA 449 F5, NETZSCH, Selb, Germany).

#### 3.3.3. X-ray Powder Diffraction

X-ray diffractometry was used as a method to analyze the crystalline nature through X-ray diffractograms [72]. The X-ray powder diffraction (XRD, Ultima IV, Rigaku, Tokyo, Japan) was operated in a step scan mode with a current of 40 mA, a voltage of 40 kV, and CuKalfa radiation (λ = 1.5416 Å). All samples were scanned from 2θ = 10° to 45° with a step size of 0.02° and a step time of 0.5 s.

#### 3.3.4. Raman Spectra

Raman spectra of the samples (lyophilized powder obtained using a freeze dryer) were obtained using a 532 nm solid-state diode laser in the spectral range of 900–1700 cm^−1^. The measurements were performed using a confocal Raman spectrometer (inVia Plus, Renishaw, London, UK).

### 3.4. Solubility Determination

Saturation solubility was determined to compare the solubility of coarse QUE, physical mixtures, and QUE-NCs. Excess samples (containing an equivalent amount of QUE) were added to conical flasks containing pure water to form supersaturated solutions. The conical flasks were placed in a constant-temperature shaker at 37 °C and oscillated at 100 rpm for 24 h. Samples were taken from the supernatants and filtered to measure the QUE content using high-performance liquid chromatography (HPLC).

### 3.5. In Vitro Drug Release

The in vitro release of 50-QUE-NCs (50 nm quercetin-nanocrystals), 140-QUE-NCs (140 nm quercetin-nanocrystals), and 360-QUE-NCs (360 nm quercetin-nanocrystals) was conducted using the dialysis method. QUE and physical mixtures were used as controls. New dialysis bags were first boiled in purified water for 10 min and then stored in purified water at 4 °C. 1 mg of drug was added to each dialysis bag (molecular interception of 8000–14,000 Da, Micro-G Biotech Co., Beijing, China). Each dialysis bag was suspended in 100 mL of release medium, which contained ethanol (40%, *v*/*v*) [73], and stirred slowly at 100 rpm at a constant temperature of 37 °C ± 0.5 °C. During stirring, 1 mL of release medium was taken at regular intervals, and 1 mL of the same release medium was added back. The collected samples were analyzed using HPLC.

### 3.6. Antioxidant Study of QUE-NCs In Vitro

#### 3.6.1. PTIO Radical Scavenging Assay

The PTIO radical scavenging ability of three QUE-NCs was assessed using the method described by Li, X (2017) with some modifications [37]. Coarse QUE, physical mixtures, and QUE-NCs of three different particle sizes were diluted to different concentrations (0.5, 0.3, 0.2, 0.1, and 0.05 mg/mL), with Vitamin C (VC) used as a positive control. The reaction solution was mixed in a 96-well plate, with 40 μL of pure water and 160 μL of PTIO (Titan, Shanghai, China) solution (0.2 mg/mL) as A_0_, 40 μL of the samples and 160 μL of PTIO solution as A_1_, and 40 μL of the samples and 160 μL of pure water as A_2_. After incubation at 37 °C for 30 min, the absorbance was measured at 557 nm using a microplate reader (EPOCH2NS-SN, Bio Tek, Winooski, VT, USA).

PTIO radical scavenging rate (%) = [1 − (A_1_ − A_2_)/A_0_] × 100%

#### 3.6.2. DPPH Radical Scavenging Assay

The DPPH radical scavenging ability of three QUE-NCs was measured following the method described by Liu F et al. (2014) with some modifications [74]. Briefly, preparations of the samples were performed as described in the PTIO radical scavenging assay above. The reaction solution was mixed in a 96-well plate, with 40 μL of pure water and 160 μL of DPPH (Macklin, Shanghai, China) solution (0.1 mM) used as A_0_, 40 μL of the samples and 160 μL of absolute ethanol used as A_1_, and 40 μL of the samples and 160 μL of DPPH solution used as A. After incubation at 37 °C for 30 min, the absorbance was measured at 516 nm using a microplate reader.

DPPH radical scavenging rate (%) = [1 − (A − A_1_)/A_0_] × 100%

#### 3.6.3. Hydroxyl Radical Scavenging Assay

The hydroxyl radical scavenging assay is based on the Fenton reaction principle [75]. Preparations of the samples were performed as described in the PTIO radical scavenging assay above. The reaction mixture consisted of the same 0.5 mL volume of 9 mM ferrous sulfate, 8.8 mM H_2_O_2_, the sample solutions, and 9 mM salicylic acid (prepared in 50% absolute ethanol). After heating in a water bath at 37 °C for 30 min, 200 μL of the mixture was transferred to a 96-well plate, and the absorbance (A_1_) was measured at 510 nm using a microplate reader (EPOCH2NS-SN, Bio Tek, Vermont, USA). The same volume of pure water was used as a blank (A_0_) to replace the sample solution, and the same volume of 50% absolute ethanol was used as a control (A_2_) to replace the salicylic acid solution. After incubation at 37 °C for 30 min, the absorbance was measured at 510 nm.

Hydroxyl radical scavenging rate (%) = [1 − (A_1_ − A_2_)/A_0_] × 100%

### 3.7. Toxicity Tests of QUE-NCs on the Development of Zebrafish Embryos In Vivo

The zebrafish embryos (purchased from Shanghai Fish Bio Co., Ltd., Shanghai, China) collected at 24 hpf (hours post-fertilization) were placed into a 24-well plate, with each well containing 15 zebrafish embryos. Each well was filled with 2 mL of nanosuspension containing 50-QUE-NCs, 140-QUE-NCs, and 360-QUE-NCs at different concentrations, respectively. The coarse QUE and physical mixture groups were also included in the test. The zebrafish embryos exposed to the E3 medium were used as the control group. Fifteen embryos were replicated for each concentration and cultured in an artificial climate incubator (RGLC-P160A, Darth Carter, Hefei, China) at 28.5 °C with a 14/10 h light/dark cycle. After 24 h, the survival rate of zebrafish embryos was counted using a stereomicroscope (SRZ-7045DM, COSSim, Beijing, China), and the embryos was then transferred to the E3 medium [50]. The developmental status of the zebrafish was observed at 96 hpf using a stereomicroscope and an inverted fluorescence microscope (Carl Zeiss AG, Oberkochen, Germany). Their heart rate was recorded at 60 s, and lateral photographs were taken to measure body length and calculate the yolk sac volume.

### 3.8. Transport of QUE-NCs in Zebrafish Embryos In Vivo

An aggregation-induced emission probe, HPS [76], was used to label the QUE-NCs and assess their uptake behavior in zebrafish embryos. HPS was dissolved in ethyl acetate and added to QUE (X_HPS_:X_QUE_ = 1:10) to prepare the HPS-QUE-NCs. Zebrafish embryos were separately exposed to QUE-NCs of three particle sizes (53 μg/mL) for 1 h. Then, they were rinsed with E3 medium and transferred into a 24-well plate. Embryos were collected at regular intervals and imaged using an inverted fluorescence microscope to observe the transport of QUE-NCs [49].

### 3.9. Effects of QUE-NCs on Antioxidant Capacity by H_2_O_2_-Induced Oxidative Stress in a Zebrafish Model

#### 3.9.1. Estimation of the Effects of QUE-NCs on ROS Generation, Lipid Peroxidation, and Cell Death in Zebrafish Embryos

The generation of ROS in zebrafish embryos was detected using an oxidation-sensitive fluorescent probe dye, DCFH-DA (Aikon, Nanjing, China) [22]. Lipid peroxidation was measured with DPPP (Titan, Shanghai, China), a non-fluorescent compound that reacts with hydroperoxides to produce a product that fluoresces strongly [21]. Cell death was measured in living embryos using acridine orange staining (Macklin, Shanghai, China) [77]. The zebrafish embryos collected at 24 hpf were transferred to a 24-well plate (15 zebrafish embryos per well) and incubated at 28.5 °C with 2 mL of nanosuspension containing 50-QUE-NCs, 140-QUE-NCs, and 360-QUE-NCs, respectively (53 μg/mL, the coarse QUE and physical mixture groups were also examined). One hour later, 5 mM H_2_O_2_ was added to the medium to induce oxidative stress, as it has the ability to diffuse across cell membranes through specific carriers and convert into highly reactive hydroxyl radicals [78]. When the embryos developed up to 96 hpf, the zebrafish were treated with DCFH-DA, DPPP, and AO and incubated for the required reaction time in the dark at 28.5 °C. Subsequently, the stained zebrafish were placed under an inverted fluorescence microscope to capture fluorescence photographs, and their fluorescence intensity was quantified using ImageJ (V.1.49, NIH, Bethesda, MA, USA). Additionally, the results of each drug treatment group were expressed as the percentage change compared to the control medium.

#### 3.9.2. Estimation of the Effects of QUE-NCs on the Activity of Antioxidant Enzymes in Zebrafish

The activity of antioxidant enzymes in zebrafish was determined following the method described by Issac P K et al. (2021) with slight modifications [54]. Forty embryos at 24 hpf were transferred to a 24-well plate containing QUE-NCs of each particle size (53 μg/mL, coarse QUE, and physical mixture groups were also examined) and incubated in an artificial climate incubator at 28.5 °C. One hour later, 5 mM H_2_O_2_ was added to the medium. After 24 h of exposure, embryos per particle size were rinsed with E3 medium and then placed into ice-cold PBS (pH 7.4) at a ratio of embryos: PBS = 1:9 (*w*/*v*) to create a 10% homogenate. The 10% homogenate was then centrifuged at 12,000 rpm for 10 min at 4 °C. Next, the supernatants were used to determine antioxidant-related parameters using CAT, GPx activity, and MDA content kits. All operations were carried out strictly following the instructions.

### 3.10. HPLC Analysis

QUE was detected at 360 nm using an HPLC system (Agilent 1100, Santa Clara, CA, USA). Separation was performed on a Welchrom Vantage C18 column (Ultimate, 250 mm × 4.6 mm, 5 μm) with a mobile phase of methanol-0.15% phosphoric acid (50:50, *v*/*v*), and the flow rate was set at 1 mL/min. In the range of 0.7 to 70 μg/mL, the QUE concentration (C) showed linearity with its peak area (A), following a typical calibration curve equation of A = 35.854C + 63.522, with an R^2^ value of 0.9984.

### 3.11. Statistical Analysis

All the measurements were performed in triplicate. One-way and Two-way analysis of variance (ANOVA) was used to compare the mean values of each treatment in GraphPad Prism software version 8.0. The survival graph was presented as a Kaplan–Meier curve and was statistically analyzed using the log-rank (Mantel-Cox) test. Significant differences between the means were identified by Tukey’s regression test. Significance was indicated in the figure as * *p* < 0.05, ** *p* < 0.01, and *** *p* < 0.001. ‘NS’ indicated that there were no significant differences.

## 4. Conclusions

To summarize, we described the successful synthesis of new antioxidant NCs fabricated by QUE through a series of stability and characterization tests. In this study, three different particle sizes of QUE-NCs, small, medium, and large, were produced to investigate the effects of size differences in vitro and in vivo. The small-sized NCs, 50-QUE-NCs, showed superiority over coarse QUE, physical mixtures, and large particle-sized NCs in vitro antioxidant and aqueous solubility studies. However, there was an adverse effect of 50-QUE-NCs in vivo, lowering the heart rate of zebrafish even at low concentrations. This issue needs further consideration regarding safety and the balance between the positive and negative effects of particle size. Additionally, QUE-NCs of all three particle sizes demonstrated significant protective effects on the survival rate of H_2_O_2_-treated zebrafish at 72 h. Conversely, the coarse QUE and physical mixture groups exhibited a survival rate equivalent to the hydrogen peroxide group, indicating no additional protective benefits compared to the H_2_O_2_ treatment alone. Moreover, small-sized QUE-NCs presented a better effect than large-sized QUE-NCs during the initial period, which could be interpreted as better permeability of small particle size. This permeability could be verified in the transport experiment.

In studying antioxidants and the enzymatic antioxidant system in zebrafish, QUE-NCs of all particle sizes displayed significant protective abilities compared to the coarse QUE and physical mixture groups, effectively restoring zebrafish to a normal state under oxidative stress. Additionally, we investigated the transport of QUE-NCs of three particle sizes, and small particle sizes of NCs indicated greater permeability to the chorion than medium and large sizes in zebrafish embryos. These results suggested that nanocrystallization of QUE could effectively exploit the antioxidant effects, and zebrafish was considered a valid model to evaluate the antioxidant effects of QUE-NCs. Furthermore, we observed that the influence of particle size on antioxidant effects was inconsistent between in vivo and in vitro settings. This finding complemented our understanding of the impact of nanocrystal particle size on antioxidants in living organisms and served as a foundation for future investigations targeting the diverse effects of various particle-sized nanocrystals in vivo.

## Data Availability

Data are available from corresponding authors on request.

## Supplementary Materials

The following supporting information can be downloaded at: https://www.mdpi.com/article/10.3390/ph16091209/s1, Table S1: Variations of average particle sizes, PDI, and zeta potential of QUE-NCs suspensions; Table S2: Preparation processes of different QUE-NCs; Figure S1: Survival rates and lateral photographs of zebrafish incubated in (A) coarse QUE suspensions, (B) 50-QUE-PM, (C) 140-QUE-PM, and (D) 360-QUE-PM; Figure S2: The photographs of DCFH-DA, DPPP, and AO staining of zebrafish from coarse QUE-, 50-QUE-PM-, 140-QUE-PM-, and 360-QUE-PM-treated groups.

## Abbreviations

AIE	Aggregation-induced emission
AO	Acridine orange
CAT	Catalase
DCF	2,7-dichlorofluorescein
DCFH	2,7-dichlorodihydrofluorescein
DCFH-DA	2′,7′-dichlorodihydrofluorescein Diacetate
DLS	Dynamic light scattering
DPPH	2,2-diphenyl-1-picrylhydrazyl
DPPP	1,3-bis (diphenylphosphino) propane
GPx	Glutathione peroxidase
GSH	Glutathione
HPS	1,1,2,3,4,5-hexaphenylsilacyclopenta-2,4-diene
Hpf	Hours post-fertilization
NCs	Nanocrystals
PTIO	2-Phenyl-4,4,5,5-tetramethylimidazoline-1-oxyl 3-oxide
PVP	Polyvinyl pyrrolidone
QUE	Quercetin
QUE-NCs	Quercetin nanocrystals
RNS	Reactive nitrogen species
ROS	Reactive oxygen species
VC	Vitamin C
50-QUE-NCs	50 nm quercetin-nanocrystals
140-QUE-NCs	140 nm quercetin-nanocrystals
360-QUE-NCs	360 nm quercetin-nanocrystals
50-QUE-PM	50 nm quercetin-physical mixture
140-QUE-PM	140 nm quercetin-physical mixture
360-QUE-PM	360 nm quercetin-physical mixture
