# Supplementary material for: Antioxidant Effects of Quercetin Nanocrystals in Nanosuspension against Hydrogen Peroxide-Induced Oxidative Stress in a Zebrafish Model
Source: Pharmaceuticals (Basel). 2023 Aug 25;16(9):1209. doi: 10.3390/ph16091209 (PMC10536595; doi:10.3390/ph16091209)
Supplement: Supplementary file 1 [file pharmaceuticals-16-01209-s001.zip › pharmaceuticals-2549447-supplementary.pdf]

Antioxidant Effects of Quercetin Nanocrystals in Nanosuspension against Hydrogen Peroxide-induced Oxidative Stress in a Zebrafish Model

Junjie Wang [1,2], Xinyue Xue [1] and Xiaoqing Miao [1,*]

[1] Marine College, Shandong University, Weihai, Shandong, 264209, China
[2] SDU-ANU Joint Science College, Shandong University, Weihai, Shandong, 264209, China
* Correspondence: xiaoqingmiao@sdu.edu.cn

Supplementary Materials

Overview

Table S1. Variations of average particle sizes, PDI, and zeta potential of QUE-NCs suspensions.

Table S2. Preparation processes of different QUE-NCs.

Figure S1. Survival rates and lateral photographs of zebrafish incubated in (A) coarse QUE suspensions, (B) 50-QUE-PM, (C) 140-QUE-PM, (D) 360-QUE-PM.

Figure S2. The photographs of DCFH-DA, DPPP, and AO staining of zebrafish from coarse QUE-, 50-QUE-PM-, 140-QUE-PM-, and 360-QUE-PM-treated groups.

Table S1. Variations of average particle sizes, PDI, and zeta potential of QUE-NCs suspensions.

Types	Time (day)	Average particle size (nm)	PDI	Zeta potential (mV)
Small (50 nm)	1	51.3 ± 0.1	0.184 ± 0.016	-16.4 ± 2.3
	2	99.9 ± 0.9	0.222 ± 0.003	-14.9 ± 1.0
	3	158.0 ± 2.5	0.274 ± 0.010	-14.1 ± 2.7
	4	253.1 ± 5.7	0.319 ± 0.036	-16.3 ± 0.9
	5	179.8 ± 5.0	0.211 ± 0.015	-18.5 ± 3.2
	6	Micro precipitation	/	0.1 ± 0.2
	8	Mass precipitation	/	0.1 ± 0.1
Medium (140 nm)	1	139.0 ± 0.6	0.068 ± 0.012	-15.1 ± 0.7
	2	195.6 ± 3.8	0.149 ± 0.013	-11.0 ± 0.4
	3	251.6 ± 4.4	0.188 ± 0.029	-15.3 ± 2.0
	4	247.7 ± 4.9	0.205 ± 0.010	-14.5 ± 0.7
	5	250.9 ± 8.6	0.171 ± 0.013	-16.5 ± 0.2
	6	Mass precipitation	/	-0.1 ± 0.2
Large (360 nm)	1	358.9 ± 4.7	0.168 ± 0.022	-13.6 ± 0.6
	2	394.0 ± 0.8	0.214 ± 0.056	-10.6 ± 2.5
	3	449.1 ± 2.4	0.119 ± 0.030	-9.0 ± 0.6
	4	518.4 ± 1.3	0.241 ± 0.004	-9.9 ± 0.9
	5	624.4 ± 14.1	0.334 ± 0.041	-8.5 ± 0.1
	6	850.0 ± 34.0	0.337 ± 0.057	-7.5 ± 0.0
	7	Mass precipitation	/	0.0 ± 0.1

Table S2. Preparation processes of different QUE-NCs.

QUE-NCs	Small particle size (50 nm)	Medium particle size (140 nm)	Large particle size (360 nm)
Formulation:	Organic phase: 1 mL of 10 mg/mL QUE ethanol solution; Aqueous phase: 1 mL of 20 mg/mL PVP mixed with 18 mL of water.	Organic phase: 2 mL of 10 mg/mL QUE ethanol solution; Aqueous phase: 3 mL of 20 mg/mL PVP mixed with 15 mL of water.	Organic phase: 1 mL of 10 mg/mL QUE ethanol solution; Aqueous phase: 3 mL of 20 mg/mL PVP mixed with 6 mL of water.
Preparations process:	The organic phase was injected into the aqueous phase at 1000 rpm at 6 mL/min (5 s).	The organic phase was injected into the aqueous phase at 1000 rpm at 6 mL/min (5 s).	The organic phase was injected into the aqueous phase at 1000 rpm at 6 mL/min (5 s).

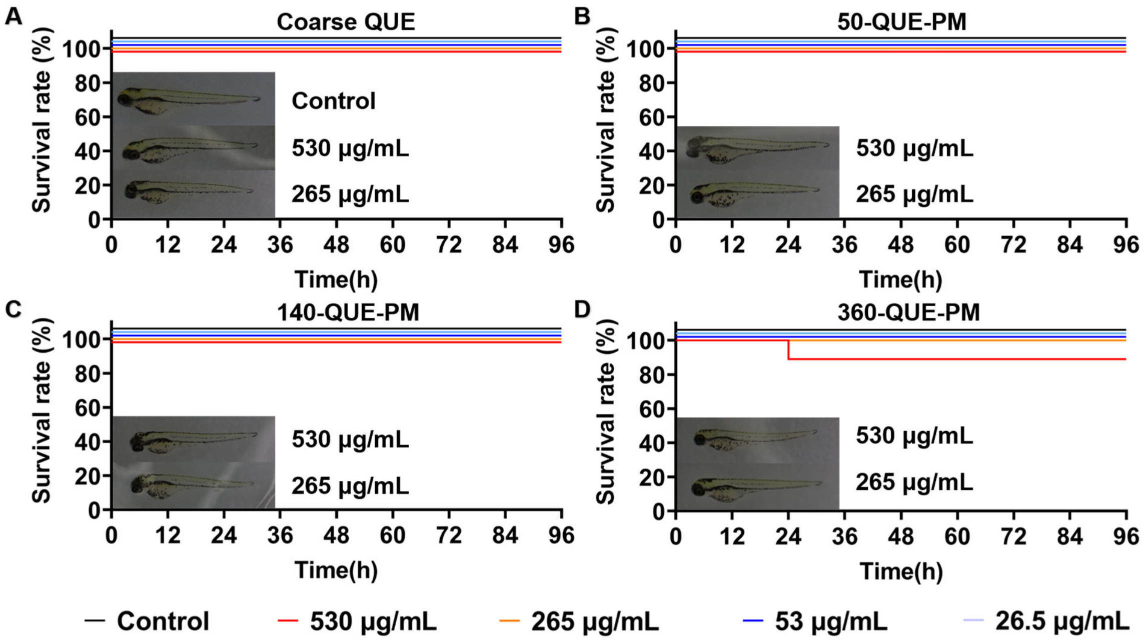

Figure S1. Survival rates and lateral photographs of zebrafish incubated in (A) coarse QUE suspensions, (B) 50-QUE-PM, (C) 140-QUE-PM, and (D) 360-QUE-PM.

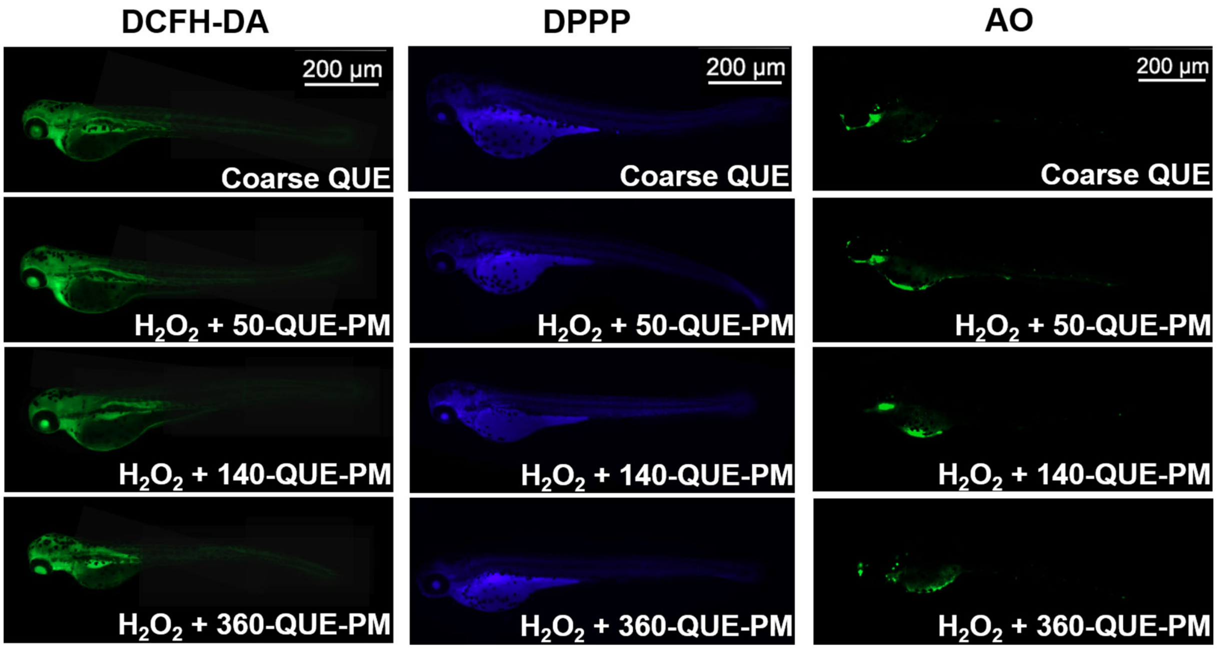

Figure S2. The photographs of DCFH-DA, DPPP, and AO staining of zebrafish from coarse QUE-, 50-QUE-PM-, 140-QUE-PM-, and 360-QUE-PM-treated groups.
